# Microbial threads in the tapestry of rheumatoid arthritis

**DOI:** 10.1172/JCI195374

**Published:** 2025-09-16

**Authors:** Jing Li, Kristine A. Kuhn

**Affiliations:** 1 Division of Rheumatology, Department of Medicine, University of Colorado Anschutz Medical Campus, Aurora, Colorado, USA.; 2 Department of Rheumatology and Immunology, Peking University People’s Hospital, Beijing, China.

## Abstract

Rheumatoid arthritis (RA) has a preclinical period of 5–10 years preceding the appearance of joint pain and swelling characteristic of clinical RA. Preclinical RA has been characterized by circulating IgA and IgG classes of autoantibodies targeting citrullinated protein antigens (ACPAs) that are highly specific for future clinical RA, circulating IgA plasmablasts, and autoantibody production at mucosal sites, all of which point toward mucosal tissues as the origin of immune dysregulation. In individuals at risk for developing and with established RA, oral and gut microbial shifts correlate with immune activation. Specific bacterial taxa such as *Segatella copri*, *Subdoligranulum didolesgii*, *Eggerthella lenta*, and Streptococcal species have been shown to contribute to the development and/or perpetuation of RA through mechanisms that include molecular mimicry, antigen citrullination, and disruption of mucosal immunity. Furthermore, microbial metabolites, including short-chain fatty acids, bile acids, and tryptophan derivatives, regulate immune homeostasis and offer potential therapeutic avenues. The gut microbiome also influences therapeutic responses by modulating conventional disease-modifying antirheumatic drugs. This Review synthesizes current knowledge on the bacterial microbiome’s role in RA pathogenesis and treatment responses, highlighting microbiome-targeted interventions as potential strategies for disease prevention and management.

## Introduction

Rheumatoid arthritis (RA) is a chronic autoimmune disorder affecting approximately 1% of the global population and characterized by persistent synovial inflammation, progressive joint damage, and systemic manifestations (1). Studies focused on the etiologies of RA have identified a prolonged period of asymptomatic autoimmunity that precedes classifiable seropositive RA. Levels of antibodies against citrullinated protein antigens (ACPAs) and inflammatory cytokines involved in RA pathogenesis, such as TNF and IL-6, increase as an individual transitions to clinical disease (2, 3). Pathogenic autoantibodies, including ACPAs, precede clinically apparent disease by an average of 3–5 years (4, 5). Approximately 30%–50% of ACPA^+^ individuals develop clinical RA within 3–5 years of follow-up (4, 5), allowing identification of a critical “at-risk” period to study the transition from at-risk to clinical disease (6).

The mucosal origin hypothesis proposes that antibodies against ACPAs, and even RA itself, begin at one or more mucosal sites, potentially triggered by microbial exposure. Intestinal dysbiosis, IgA isotype ACPAs, expanded circulating IgA plasmablasts during the at-risk phase of RA, and the presence of mucosal ACPAs support a mucosal origin of RA (7). Many have sought to identify microbial triggers, and several candidate bacteria have been identified through studies of the microbiome.

The role of the microbiome in RA pathogenesis spans several critical aspects. Specific microbial taxa in the gut and oral cavity are increasingly implicated in triggering autoimmune responses, particularly in genetically susceptible individuals. Furthermore, microbiome changes, often described as dysbiosis, are not only associated with disease onset but also appear to perpetuate inflammation during disease progression (8, 9). The influence of the microbiome extends to therapy, where its composition may affect the efficacy of treatments such as conventional disease-modifying antirheumatic drugs (cDMARDs) and biologic therapies (10). Understanding these interactions provides a foundation for novel predictive, diagnostic, and therapeutic approaches.

This Review aims to synthesize current knowledge on the microbiome’s role in RA, focusing on three key areas: the microbiome’s characteristics in at-risk and established RA, including changes induced by treatment and its impact on therapeutic efficacy; mechanisms through which the microbiome can initiate and sustain autoimmunity; and finally, insights for leveraging the microbiome in the management of RA. By exploring these dimensions, this Review highlights the microbiome as a potential contributor to RA pathogenesis and a promising target for innovative interventions.

## Microbiome in individuals at risk for and with established RA

**Figure 1:**
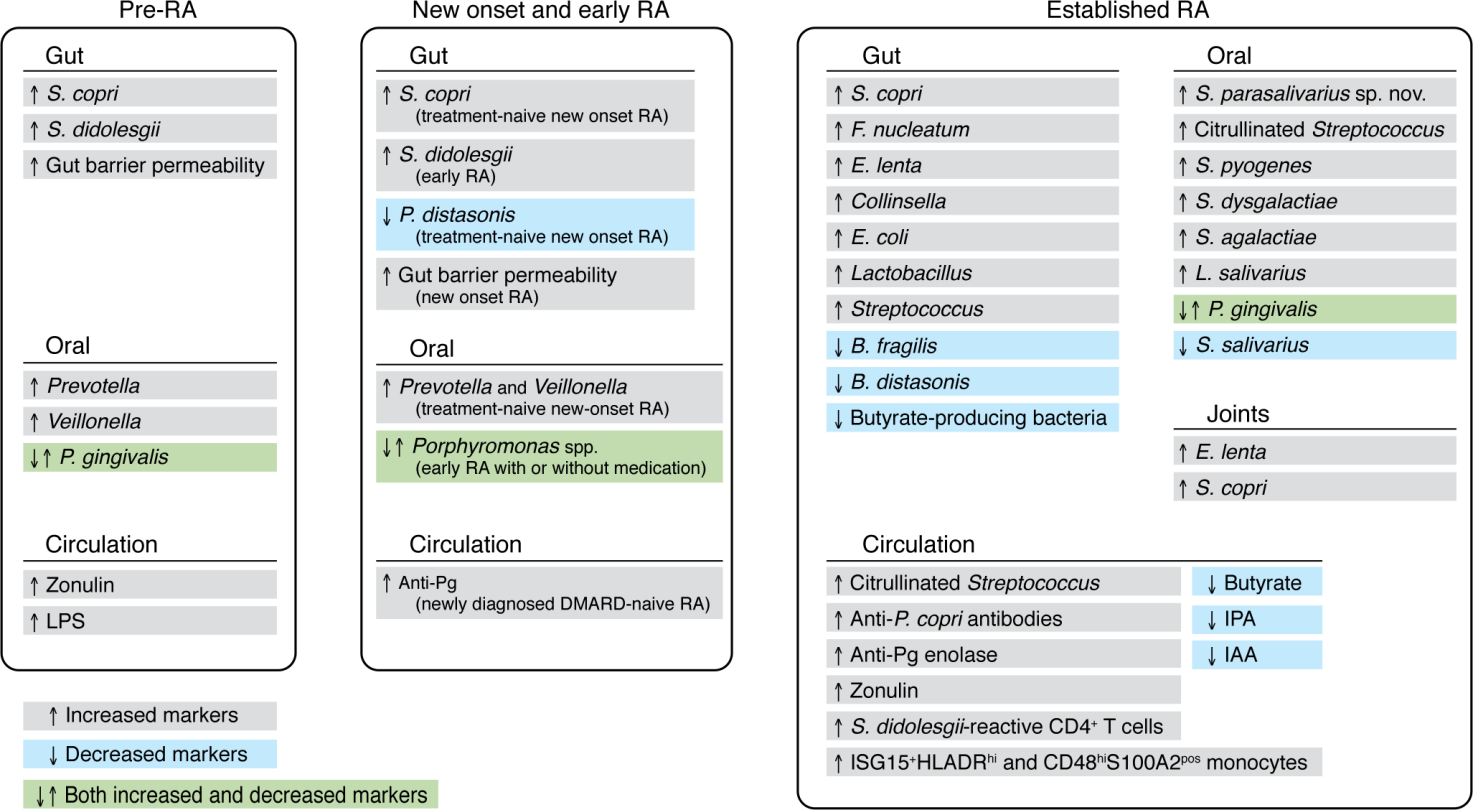
Microbial and immune changes across the stages of rheumatoid arthritis. Changes in the composition of gut and oral microbiota as well as systemic responses observed in three stages of rheumatoid arthritis (RA): preclinical (Pre-RA), new-onset and early RA, and established RA. Pg, *P*. *gingivalis*; IPA, indole-3-propionic acid; IAA, indole-3-acetic acid; DMARD, disease-modifying antirheumatic drug. Black text indicates increased markers, blue text indicates decreased markers, and green text indicates markers that show both increases and decreases.

Emerging evidence suggests that changes in microbiota begin prior to clinical disease and further change with treatment of RA. Although studies identify numerous microbial candidates at oral and intestinal sites, some patterns emerge (Figure 1). However, data to date focus primarily on bacteria, with little information regarding viruses and fungi; thus, we focus our Review on the bacterial microbiome in RA, with brief mention of data associating *Candida* species and viruses.

### Bacterial dynamics in individuals at risk for RA.

Most studies define “at risk for RA” as individuals who are serum ACPA^+^ but without typical clinical features of RA such as synovitis on exam. In such individuals, *Prevotella* spp., particularly *Segatella* (formerly *Prevotella*) *copri*, are consistently enriched in the stool (11, 12). Host genetics further influence gut microbiota composition in this context, as supported by the finding that a polygenic risk score for RA positively associated with the presence of *Prevotella* spp., in the absence of clinical symptoms (13). This finding suggests a bidirectional relationship in which RA-related genetic variants may shape the gut microbiome, while microbial dysbiosis, in turn, may contribute to immune activation and disease progression. Supporting this conclusion, a recent prospective analysis of serum ACPA^+^ individuals with musculoskeletal symptoms who ultimately developed RA revealed significant fluctuations in the microbial community composition, especially *Prevotella* spp., that occurred approximately 10 months before RA onset. This finding is in comparison with a control population of individuals with serum ACPA^+^ and musculoskeletal symptoms who did not develop RA during a 12-month follow-up period (12). This instability may signify a late microbial shift that contributes to RA progression.

Beyond gut microbiota, numerous studies have reported changes in the oral microbiomes of individuals at risk for RA. An increased abundance of *Prevotella* and *Veillonella* has been observed (14, 15). However, the presence of oral *P*. *gingivalis* is inconsistent, with some studies reporting an increase (16) and others a decrease (15, 17) in prevalence, although these discrepancies may be influenced by variations in study cohorts, collection methods, geography, genetic predisposition, and other contributing factors.

### Bacterial changes in individuals with established RA.

Cross-sectional studies evaluating the oral and gut microbiomes from individuals with RA have associated numerous bacteria with disease. Machine learning approaches have demonstrated that fecal, dental, and oral microbiomes from individuals with RA can distinguish disease with high accuracy (AUCs of 0.93, 0.87, and 0.81, respectively) (18), although these findings require validation in independent cohorts.

Like individuals at risk for RA, *Prevotella* spp., particularly *S*. *copri*, is consistently detected in the stool from individuals with established disease (12, 19, 20). Notably, it is also found in synovial tissue, but its role in pathophysiology at this site is unknown (20, 21). The pathogenic potential of *S*. *copri* is associated with genomic adaptations, particularly the acquisition of conjugative transposons that enhance its ability to modulate host innate immune responses (22).

Similarly, products from *Fusobacterium nucleatum* and *Eggerthella lenta*, enriched in the stool from patients with RA, have been identified in the synovial fluid of patients with RA and associated with markers of inflammation and measures of disease severity (20, 23–25). However, whether and how the presence of bacteria in the synovium and synovial fluid affect RA is unknown. Expansions of *Collinsella* (stool), *Escherichia coli* (stool), and *Lactobacillus* spp., particularly *L*. *salivarius* (stool, oral) also associate with RA and clinical factors such as autoantibodies and systemic inflammatory cytokines (18, 20, 26–28). Conversely, some *Lactobacillus* spp. like *L*. *salivarius* strain UCC118 and *L*. *plantarum* WCFS1 (stool), as well as *Parabacteroides distasonis* (stool), are reduced in abundance and may be protective in RA through associated antiinflammatory mechanisms such as inducing IL-10 or suppressing Th17 cells (29, 30). The abundance of periodontal and oral bacteria, like *Porphyromonas gingivalis*, varies across studies, with some reporting an increase (31), others a decrease (17, 18), and some showing no significant change (32–34). The abundance of *P*. *gingivalis* may be more strongly linked to periodontitis severity rather than being specific to RA (33).

The tonsillar microbiota in individuals with RA is enriched for pathogenic species such as *S*. *pyogenes*, *S*. *dysgalactiae*, and *S*. *agalactiae*, with experimental models demonstrating their role in exacerbating arthritis severity by promoting immune cell activation and inflammatory responses. Conversely, the depletion of protective species such as *S*. *salivarius* in patients with RA contributes to microbial imbalance and immune dysregulation (35). The loss of *S*. *salivarius* is particularly significant, as this species produces salivaricins, which are lantibiotic peptides with immunomodulatory properties (36, 37) that inhibit T follicular helper (Tfh) cell differentiation and IL-21 production, thereby reducing antibody production and systemic autoimmunity (37). Notably, supplementation with *S*. *salivarius* or its salivaricins in murine models effectively attenuates arthritis progression (35, 37).

### Bacterial changes in response to treatment.

RA treatments also actively reshape the gut microbiome, leading to microbial shifts that may influence disease progression and treatment outcomes (10, 38). Immunomodulatory therapies, including DMARDs and biologics, have been shown to partially restore gut dysbiosis in patients with RA, with microbial shifts correlating with improved clinical outcomes and reduced disease activity. However, these effects are often incomplete, as patients with longstanding RA retain a distinct microbiota composition compared to healthy individuals despite prolonged treatment (18).

Among conventional DMARDs, methotrexate (MTX) treatment did not significantly alter overall gut microbiota composition (39, 40) but induced significant shifts in gene family abundance, particularly in pathways related to pyrimidine synthesis, protein synthesis, and ABC transporters (40). Hydroxychloroquine may similarly contribute to microbiota modulation by suppressing proinflammatory bacterial overgrowth (41), although data on its role in RA microbiome effects are scarce. Biologic therapies, including TNF inhibitors such as etanercept, also influence gut microbial composition. TNF inhibitor treatment in patients with RA partially restores gut microbiota composition by increasing beneficial bacterial taxa and reducing dysbiosis-associated changes, with notable modulation of *Euryarchaeota*, which correlates with disease severity (39).

### Fungal and viral microbiomes in RA.

The fungal and viral microbiomes in RA have been studied less extensively compared with bacteria. Fungi constitute only a minor fraction of the intestinal microbiota — typically accounting for 0.1% to 1.0% (42, 43). Among gut fungi, *Candida albicans* is a predominant member of the intestinal mycobiome, recognized both as a commensal organism and an opportunistic pathogen (42, 43). Relative abundances of *Candida* species are increased in the fecal microbiota of individuals with RA (44, 45), and colonization of mice by *C*. *albicans* can worsen disease in murine arthritis (46, 47). Notably, β-glucan, a structural component of the *C*. *albicans* cell wall, acts as an immunological adjuvant capable of promoting autoimmune arthritis in mice (48–50).

The intestinal virome in individuals at risk for developing RA compared with controls is enriched with Streptococcaceae, Bacteroidaceae, and Lachnospiraceae phages, which associated with cyclic citrullinated peptide (CCP) positivity (51). Interestingly, a phage-encoded phosphonate phosphodiesterase that associated with CCP-positive at-risk individuals (51), is an ortholog of a gene that associated with predicted response to MTX therapy in another study (52). Within individuals with established RA, crAss-like phages are significantly reduced compared with controls (53), although when examined by treatment status, family *Phycodnaviridae* were significantly decreased in treated patients (54).

## Proposed microbial mechanisms leading to RA

**Figure 2:**
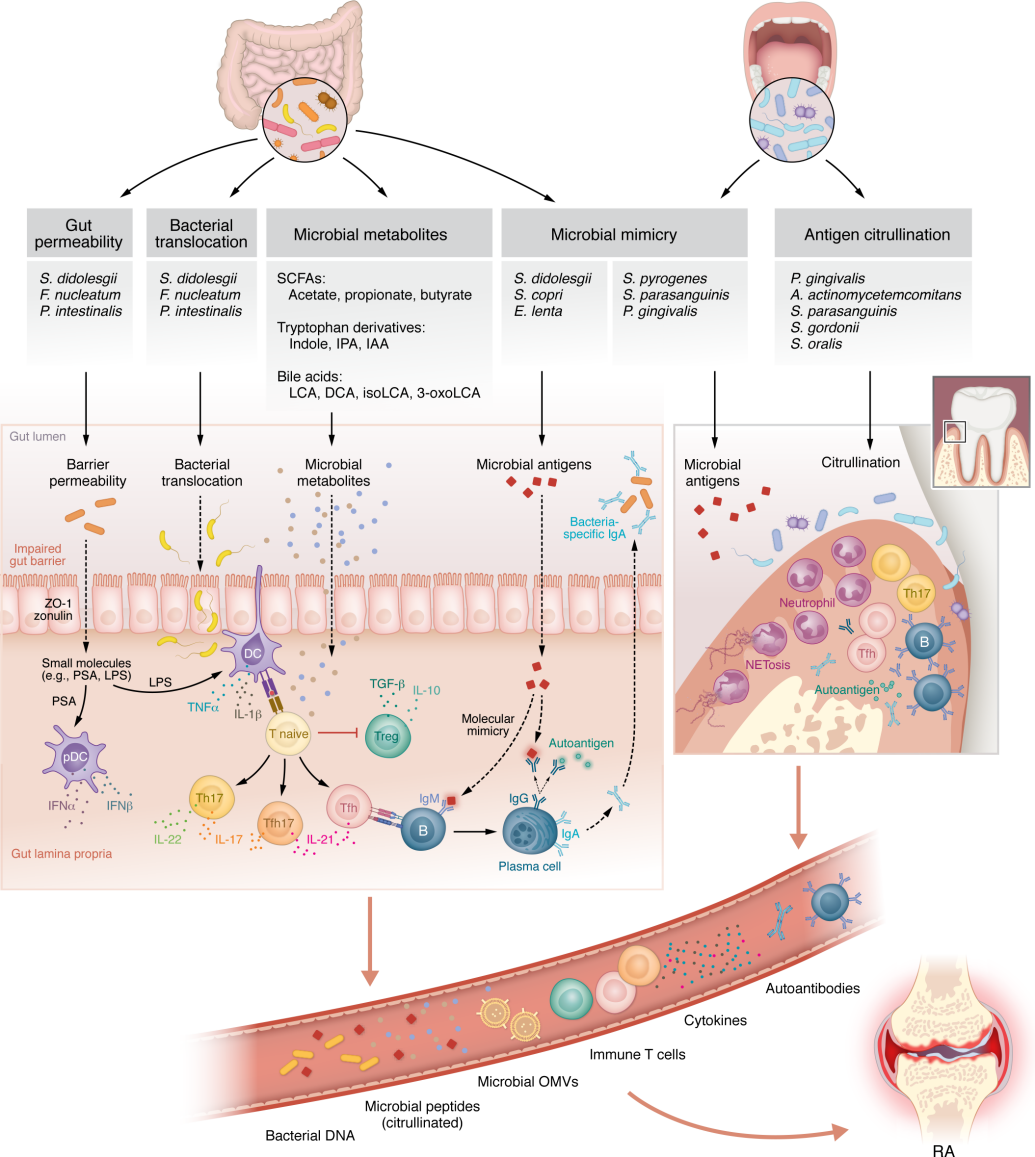
Potential mechanistic effects of the microbiome in RA pathogenesis. Bacterial dysbiosis in the gut and oral cavity promotes barrier disruption, bacterial translocation, and altered microbial metabolite production. Increased gut permeability facilitates dissemination of microbial components such as LPS and polysaccharide A (PSA), priming innate immune responses and promoting T cell polarization toward Th1, Th17, and Tfh subsets. Microbial metabolites, including short-chain fatty acids (SCFAs), tryptophan derivatives (e.g., indole, IPA, IAA), and bile acids (e.g., LCA, DCA), modulate local and systemic immune responses. Specific bacteria contribute to RA via molecular mimicry (*S*. *didolesgii*, *P*. *copri*, *E*. *lenta*) or by promoting antigen citrullination (*P*. *gingivalis*, *A*. *actinomycetemcomitans*, *S*. *parasanguinis*), leading to the generation of autoantibodies. Neutrophil activation and NETosis further expose citrullinated microbial and host antigens. The combined effects of microbial translocation, antigenic stimulation, molecular mimicry, and citrullination establish a link between mucosal microbiota and systemic autoimmunity in RA.

There are likely multiple mucosal sites, as indicated by the multiple sites of microbial dysbiosis described above, and pathways by which the microbiota can contribute to the development of RA. Among the pathways are the generation of neoantigens through citrullination, molecular mimicry, epithelial barrier permeability, microbial translocation, and microbial education of immune responses (Figure 2). These mechanisms, along with the mucosal sites where they occur, are not mutually exclusive. Multiple mechanisms and mucosal sites likely converge to lead to autoimmunity and ultimately clinical RA.

### Generation of citrullinated antigens.

Given the central role of ACPAs in the diagnosis and pathophysiology of RA (55), identifying the source of citrullinated antigens that drive ACPA production remains a key focus of investigation. Citrullination of arginine residues in proteins occurs as a posttranslational modification, catalyzed by the family of peptidyl arginine deiminases (PADs). Microbial factors, particularly bacterial PADs and microbe-induced host PAD activation, contribute to the generation of citrullinated antigens in mucosal sites. The only bacterium known to express PAD is the periodontal pathogen *P*. *gingivalis*, which can generate citrullinated fibrinogen and enolase, antigens often targeted by ACPAs (56, 57). Experimental models demonstrated that PAD-expressing strains of *P*. *gingivalis* exacerbated arthritis, whereas PAD-deficient strains failed to do so (58, 59). Conversely, some periodontal bacteria and pathogens, such as *Aggregatibacter actinomycetemcomitans*, indirectly induce citrullination by triggering host PAD activation to subvert host immunity. The resulting hypercitrullination of host proteins closely resembles the citrullinated antigens found in RA joints (60, 61). Additional bacteria like *Staphylococcus aureus* and viruses like rhinovirus and cytomegalovirus similarly induce host hypercitrullination that may result in loss of tolerance leading to RA (61). However, the importance of bacterial species, the processes of antigen citrullination, and the temporal relationship between microbial exposure and citrullination in the generation of ACPAs remain unresolved.

### Molecular mimicry.

For decades, researchers have sought to identify the elusive “arthritogenic” antigen, citrullinated or not. A leading hypothesis proposes that microbial antigens resemble host proteins that may provoke cross-reactive immune responses, ultimately breaking immune tolerance and promoting systemic autoimmunity (7). *S*. *copri*, *Subdoligranulum didolesgii*, and *Streptococcus* spp. each have promising data supporting molecular mimicry that leads to RA.

Initial studies to identify potential microbes that could serve as molecular mimics of self-antigens utilized HLA-DR peptidomics. Peptides presented by HLA-DR on cells from the peripheral blood, synovial tissue, and synovial fluid from individuals with RA revealed sequence homology between the *S*. *copri* protein Pc-p27 and self-antigens, including N-acetylglucosamine-6-sulfatase (GNS) and filamin A (FLNA), both of which are expressed in RA synovial tissues. Pc-p27 elicited robust T cell responses that cross-reacted with their human counterparts, producing a pronounced Th1 response in patients with RA, while IgA antibodies targeting Pc-p27 associated with increased levels of ACPA and Th17 cytokines (62–64). In arthritis-prone SKG mice, *S*. *copri* colonization promoted Th17 expansion, joint inflammation, and autoantibodies. Furthermore, *S*. *copri*–educated T cells from SKG mice could trigger arthritis when transferred to naive T cell–deficient mice (65), supporting a causal role for cross-reactive immune responses in driving autoimmune arthritis.

In another approach to identify possible molecular mimics, autoreactive monoclonal antibodies (mAbs) derived from patients at risk for and with RA were used to identify possible cross-reactive bacteria in a pool of fecal samples. Through 16S sequencing of bacteria bound to the mAbs, followed by culturing bacteria from the primary fecal samples, *Subdoligranulum didolesgii* emerged as a candidate molecular mimic. In addition to being targeted by the autoreactive mAbs, *S*. *didolesgii* specifically activated T cells with a Th17 phenotype in a MHC class II–dependent manner from individuals with RA and not controls. To demonstrate causality, colonization of germ-free mice with *S*. *didolesgii* induced joint inflammation, Th17 activation, and pathogenic autoantibody production as evidenced by the ability of serum from *S*. *didolesgii* colonized mice to transfer arthritis (66). Although the specific T and B cell antigens are yet to be identified, these findings highly suggest that *S*. *didolesgii* may be another trigger for molecular mimicry in RA.

As a third approach to identify molecular mimics, paired bacterial and human transcriptomics of blood from individuals with concomitant periodontitis and RA demonstrated systemic translocation of citrullinated oral *Streptococcus* spp. from the oral mucosa preceding an RA flare. Inflammatory ISG15^+^HLA-DR^hi^ and S100A12^+^ monocytes and antibody effector response transcripts also associated with RA flare, suggesting that the citrullinated *Streptococcus* could trigger autoantibody responses. Indeed, ACPA mAbs derived from human RA plasmablasts cross-reacted with citrullinated bacteria including *Streptococcus*, but not uncitrullinated bacteria (67), implicating citrullinated *Streptococcus* spp. as another potential molecular mimic.

These findings support molecular mimicry as one possible mechanism in RA, yet critical gaps remain. Although microbial peptides that mimic self-antigens have been suggested, their precise role in breaking immune tolerance and driving disease progression is not fully elucidated. Microbial antigens may act as initial triggers or exacerbate preexisting autoreactivity, and these antigens may act individually, sequentially, or in concert. Finally, the diversity of microbial epitopes capable of eliciting cross-reactive immune responses complicates efforts to define causative agents in RA.

### Barrier permeability.

Translocation of small microbial molecules is controlled by epithelial permeability, a tightly regulated process allowing local mucosal immune cells to respond appropriately to their environment. The molecular exchange is size-restricted and prevents passage of whole microbial cells. Thus, the colloquial term “leaky gut” should be more accurately referred to as increased intestinal permeability, a condition that permits increased translocation of microbial molecules, and not whole organisms, across the intestinal barrier.

Permeability is determined by the expression of tight junction proteins (e.g., zonula occludens-1 [ZO-1], occludin, and claudins in the intestine), which act as selective gatekeepers regulating molecular exchange between the mucosa and its lumen (68). Tight junctions are dynamically influenced by both microbial signals (25, 69, 70) and host cytokines (71–75). Microbes regulate tight junction expression through toxins, pathogen-associated molecular patterns, and metabolites (68). RA-associated gut bacteria, such as *C*. *aerofaciens* and *E*. *lenta*, reduce barrier permeability by downregulating key tight junction proteins, including ZO-1 and occludin (25, 69, 70). Although the precise signals are unknown, microbial products might engage pattern recognition receptors, including Toll-like receptors (TLRs) and nucleotide-binding oligomerization domain–containing (NOD) proteins. TLR2 signaling reduces barrier permeability by stabilizing ZO-1 (76), but TLR4 signaling increases permeability by reducing claudin-1 and ZO-1 expression via NF-κB activation (77). NOD1/2 signaling reduces barrier permeability by enhancing E-cadherin expression (78). Additionally, proinflammatory cytokines, like IL-17, TNF, and IL-1β, can be induced by some bacteria and increase barrier permeability (74, 75, 79). In contrast, certain probiotics and microbial metabolites reduce permeability. Bacteria such as *B*. *pseudocatenulatum* (80), *P*. *distasonis* (30), and *P*. *histicola* (81) increase tight junction protein expression. Some microbial metabolites such as butyrate support epithelial cell function, reduce permeability, curb inflammatory cytokine production, and maintain immune homeostasis (82, 83).

The principal consequence of increased intestinal permeability is the activation of immune cells. Increased intestinal permeability allows microbial components such as lipopolysaccharides (84), peptidoglycans (85), and outer membrane vesicles (OMVs) (23) to cross the epithelial barrier, exposing local immune cells to activating ligands. As a result, immune cells are primed for subsequent activation (86–88). For example, mice deficient in junctional adhesion molecule A (JAM-A), a critical molecule that supports tight junctions, exhibit a tenfold increase in intestinal permeability. Although T and B cells infiltrate the lamina propria more extensively in JAM-A–deficient mice, they do not spontaneously develop colitis. Rather, JAM-A–deficient mice experience a more severe clinical course in the dextran sodium sulfate colitis model (87, 89). These data suggest that increased paracellular permeability primes immune cells for easier activation, indicating that while heightened permeability does not cause inflammation under steady-state conditions, it predisposes the immune system to exacerbated responses during inflammation.

In preclinical arthritis models, intestinal permeability is often increased, associated with arthritis severity. Microbial dysbiosis in collagen-induced arthritis (CIA) and K/BxN mice corresponds with increased intestinal permeability, increased IL-17 in intestinal tissue (90), and arthritis severity (91). Transfer of the dysbiotic microbiome from CIA mice to germ-free mice resulted in increased intestinal permeability (82). Interventions that reduced intestinal permeability, such as butyrate or the zonulin antagonist larazotide acetate, inhibited arthritis development in CIA and antigen-induced arthritis (AIA) models (82, 91). These findings suggest that reducing barrier permeability may prevent or mitigate disease.

Several studies report possible increased intestinal permeability in at-risk and established RA individuals through use of indirect measures, including increased circulation of the tight junction stabilizing protein zonulin, LPS, lipopolysaccharide-binding protein (LBP), and soluble CD14 (sCD14), alongside decreased colonic expression of the tight junction protein ZO-1 (82, 92–94). However, these measures of permeability have limitations. Assays that measure zonulin have cross-reactivity with haptoglobin (95), and factors like LPS and sCD14 can be acute-phase reactants (96). Although these indirect measures of intestinal permeability are associated with RA, the mechanistic consequences are unknown. Thus, it remains unclear whether increased epithelial permeability has a pathophysiologic role.

### Microbial translocation.

Mechanisms of bacterial translocation often occur as transcellular passage through the epithelium, and not through tight junctions or mechanisms of epithelial permeability (97–100). Oral citrullinated *Streptococcus* spp. translocate across the periodontium into the circulation of patients with RA (67), and bacterial products have been detected in both synovial tissue and fluid of patients with RA, including DNA from *S*. *copri* and *E*. *lenta* (25, 69, 70), which are enriched in the gut microbiota of patients with RA (12, 19, 20, 24). For the bacterium *Fusobacterium* *nucleatum*, which is enriched in patients with RA, murine models demonstrate translocation to the joint of OMVs carrying the FadA protein, which activates synovial macrophages and promotes joint inflammation (23). *Prevotella intestinalis* and *S*. *copri* similarly make OMVs that are able to enhance CIA (101), although the site to which the OMVs translocate was not evaluated. Nevertheless, bacterial products may not need to circulate or migrate to the joint. In the case of *S*. *didolesgii*, bacterial DNA is found in the intestinal epithelium of monocolonized mice, where it triggers local IgA and Th17 immune responses (66).

### Microbial metabolites.

Microbial metabolism generates an array of immunomodulatory molecules that can influence host immune functions through multiple pathways (102–104). Metabolites such as short-chain fatty acids (SCFAs) (105), tryptophan catabolites (106, 107), and bile acids (108, 109) have been associated with RA. In addition to maintaining intestinal barrier homeostasis (80, 82, 110, 111), these microbially derived metabolites shape immune responses relevant to RA.

SCFAs, primarily acetate, propionate, and butyrate, are fermentation products of dietary fibers and are well recognized for their immunomodulatory effects (105, 112). They act through free fatty acid receptor and G protein–coupled receptors, and influence gene expression by inhibiting histone deacetylases and activating histone acetyltransferases (112). These pathways collectively promote regulatory T cell (Treg) differentiation while suppressing proinflammatory Th17 and Tfh cells (112).

Among SCFAs, butyrate is significantly depleted in patients with RA (105, 112–114), corresponding to a microbial imbalance characterized by reduced butyrate-producing bacteria and increased butyrate-consuming species (115–117). Furthermore, individuals at risk for RA who have higher serum levels of SCFAs exhibit a lower likelihood of progressing to clinical RA (115). In arthritis-induced experimental models, including CIA, SKG, and AIA, butyrate supplementation has been shown to alleviate disease severity, reduce bone erosion, and modulate immune cell populations (82, 115, 117–120). These effects are largely attributed to the promotion of Treg, T follicular regulatory (Tfr), and regulatory B (Breg) cell differentiation, alongside suppression of Tfh and Th17 cells (115, 116, 118, 119). However, in collagen antibody–induced arthritis and K/BxN serum-transfer models, models of the effector phase rather than induction phase of arthritis, butyrate does not confer therapeutic benefit (118, 120, 121). These observations suggest stage-dependent effects of butyrate in RA; increasing butyrate in preclinical disease may be therapeutic, whereas in later stages when autoimmunity or disease is established, butyrate does not have such benefits.

Tryptophan metabolism represents another key microbial pathway influencing RA. Commensal and probiotic species, including *Lactobacillus* and *Bifidobacterium*, generate tryptophan catabolites in two primary pathways: indole and kynurenine. The indole pathway is largely microbiome derived, producing metabolites such as indole, indole propionic acid (IPA), and indole acetic acid (IAA) (106, 107). In patients with RA, altered tryptophan catabolism results in reduced levels of antiinflammatory indoles like IPA and IAA, alongside increased levels of the proinflammatory quinolinic acid (122). Indole-containing compounds signal through the aryl hydrocarbon receptor (AhR) on host immune cells, mediating immune responses (123). IPA and IAA alleviate arthritis in the CIA model through increased Treg differentiation and suppression of inflammatory cytokines (124). However, certain bacteria enriched in RA, such as *Lachnospiraceae* and *S*. *didolesgii*, exacerbate RA by overproducing primary indole and stimulating IL-6, IL-1β, and IL-17 signaling (124), underscoring a dual role of tryptophan metabolism that depends on the specific microbial and host context.

Bile acids are another class of microbially modified metabolites that may also influence RA. Secondary bile acids, including deoxycholic acid (DCA) and lithocholic acid (LCA), are produced by intestinal bacteria from hepatically derived primary bile acids. These metabolites regulate metabolism, barrier permeability, immune responses, and gut microbiota composition (110, 111). Antiinflammatory secondary bile acids such as DCA and LCA primarily act through Takeda G protein–coupled receptor 5 (TGR5) signaling (80), suppressing Th17 differentiation and promoting M2 macrophage polarization to reduce systemic inflammation and CIA severity (30, 80). Finally, in a short, 14-day open-label Mediterranean diet–inspired “anti-inflammatory foods” dietary intervention, individuals with RA who achieved a 50% reduction in pain exhibited significantly higher serum bile acid levels compared with those without pain improvement (125). These data strongly suggest that secondary bile acids may be protective in the development and/or perpetuation of RA.

Despite promising preclinical evidence, translating microbial metabolite research into RA therapies remains challenging due to the complexity of host-microbiota interactions, individual variability, and context-dependent effects. Although SCFAs, bile acids, and tryptophan catabolites influence key immune processes, such as Treg differentiation and cytokine production, their dual roles in inflammation underscore the need for caution. Realizing their therapeutic promise will require deeper mechanistic insights, personalized strategies, and robust clinical validation.

## Treatment opportunities

Advancing our understanding of the microbiome’s role in RA has unveiled several therapeutic opportunities, ranging from microbial manipulation and dietary interventions to precision medicine based on microbial signatures. These strategies offer novel avenues to modulate disease activity, improve patient outcomes, and potentially predict treatment responses.

### Microbial manipulation.

Efforts to reshape the gut microbiota using prebiotics, probiotics, and fecal microbiota transplantation (FMT) have shown varying degrees of promise in RA. Prebiotics, typically high-fiber compounds intended to promote the growth of beneficial microbes, may make patients feel better but do not improve disease activity. In one double-blind, placebo-controlled trial involving 69 patients, no significant improvements in disease activity scores (DAS) were observed (126). In contrast, short-term and long-term interventions using probiotics have demonstrated reductions in inflammatory markers and joint symptoms in small double-blind placebo-controlled trials. An 8-week trial with *Lactobacillus casei* and a 12-month trial with *Lactobacillus rhamnosus* both reported decreases in swollen joints and inflammatory activity (127, 128). Similarly, a multistrain 8-week study combining *L*. *casei*, *L*. *acidophilus*, and *Bifidobacterium bifidum* led to lower levels of C-reactive protein (CRP), a circulating marker of inflammation, though paradoxically, disease activity scores increased (129). Although these findings may indicate potential for prebiotics and probiotics in helping patients with RA feel better, they emphasize the need for larger, better-conducted studies to clarify efficacy and strain-specific effects.

FMT represents a more direct approach to microbial manipulation, but has not been formally studied in RA. A case report described a 20-year-old woman with refractory seropositive RA who underwent FMT from a healthy pediatric donor. She achieved minimal disease activity within one week of FMT, maintained through a 78-day follow-up period (130); however, the conclusions are limited by the concomitant use of corticosteroids with the FMT and through the follow-up period. More formal testing of FMT has been conducted in a clinical trial for psoriatic arthritis (PsA), where 31 patients were randomized to receive either FMT or sham treatment after washout of immunosuppressive therapies. Sixty percent of the FMT group required rescue therapy within 32 days compared with 19% in the sham group, suggesting limited efficacy in this setting (131). These outcomes emphasize caution around the safety and efficacy of FMT in RA.

Emerging areas of microbial manipulation include transplantation of defined bacterial consortia and engineered microbes, approaches that are under study for inflammatory bowel diseases. VE202 composed of beneficial Clostridia strains, SER-301 with 18 Firmicutes members, and MH002 comprised of a six-member defined consortia are each being tested for the treatment of ulcerative colitis (132). Engineering microbes to manage inflammation may be another strategy for therapeutically manipulating the microbiome. Gene editing of *Saccharomyces* *cerevisiae* to sense and degrade extracellular adenosine triphosphate, which contributes to intestinal inflammation, was effective in treating mouse models of inflammatory bowel disease (133). Finally, the use of bacteriophages to target and eliminate specific microbes that contribute to inflammatory bowel disease is being explored (134). Similar approaches may be considered as potential therapeutics for RA.

### Dietary modulation.

Numerous studies have evaluated the role of diet for modulating disease activity in RA (135). Initial studies suggested that fasting (reduction of daily caloric intake) may reduce disease activity, including morning stiffness, tender and swollen joints, and CRP, which reversed when normal dietary habits resumed (136, 137). Adoption of a Mediterranean diet, versus a Western diet, also reduced disease activity for patients with RA (138). However, these studies did not consider how dietary modulations could manipulate the microbiome’s potential to mediate inflammation.

Newer dietary strategies aim to reshape the microbiome and metabolome in RA. Fiber-rich diets significantly elevate serum SCFAs, which are associated with reductions in proinflammatory cytokines (MCP-1, IL-18, IL-33), an increase in Tregs, and clinical improvement measured by the Health Assessment Questionnaire (HAQ) (139). Furthermore, as described above, an “anti-inflammatory” foods–altered Mediterranean diet may contribute to symptom improvement through increased secondary bile acids (125). Another study in which microbiome and metabolome-guided dietary intervention is underway, focusing on the Mediterranean diet with enrichment of *n*-3 polyunsaturated fatty acids, phenols, and fermented foods containing probiotics. Outcomes will include disease activity assessments as well as microbiome and metabolome profiling (140).

In addition to changes in dietary intake, altering dietary timing may be another approach. During periods of food consumption, select gut microbes, particularly *Parabacteroides distasonis*, increase in abundance. In the CIA model, *P*. *distasonis* exerted antiinflammatory effects by producing glycitein, a product of flavonoids, which in turn attenuates SIRT5-mediated inflammatory pathways. Fecal *P*. *distasonis* and serum glycitein, which are low at night and increase during the daytime in patients with RA, negatively correlated with IL-6, TNF, and other disease activity measures (141). Thus, manipulating the diurnal fluctuations of the microbiome may serve to reduce disease activity in RA.

### Predicting treatment response.

Beyond therapeutic interventions, the microbiome holds potential for guiding treatment selection. The intestinal microbiome influences drug metabolism, therapeutic efficacy, and long-term disease outcomes (10, 38, 40, 142–145). One of the most well-characterized examples of microbial involvement in drug metabolism is sulfasalazine. Sulfasalazine is an azo-bonded prodrug that requires cleavage by colonic bacterial azoreductases to release its active metabolite, 5-aminosalicylic acid (146), and sulfapyridine. The efficacy of sulfasalazine is thus contingent upon the presence and enzymatic activity of these gut bacteria, including species such as *E*. *coli*, *Enterococcus faecalis*, and *Bacillus subtilis* (147). Although not examined in RA, patients with inflammatory bowel disease associated spondyloarthritis who have *Faecalibacterium prausnitzii* enriched in their gut microbiome were more likely to respond to sulfasalazine treatment, likely through sulfapyridine-promoted butyrate production by *F*. *prausnitzii* (145).

Similarly, microbe-driven drug metabolism is observed with MTX (148, 149). Studies in germ-free and antibiotic-treated mice demonstrated that the absence of a gut microbiota results in reduced intestinal absorption and metabolism of MTX (150). MTX directly alters bacterial physiology by inhibiting bacterial dihydrate folate reductase, resulting in reduced purine and pyrimidine synthesis. As such, the composition and metabolic activity of the gut microbiome changes such that mucosal immune cells become less inflammatory (reduced Th1 and Th17 populations and increased Tregs) (40). Thus, MTX exerts part of its immunomodulatory effects through microbiome modulation (40, 151). Such changes in microbial composition may allow prediction of responders to MTX therapy. MTX responders exhibited a marked reduction in *Bacteroidetes*, while nonresponders displayed significant shifts in microbial gene functions related to nucleotide and folate metabolism (40). Furthermore, two microbiome enterotypes have been described in RA — *Prevotella*-dominant and *Bacteroides*-dominant — which correlate with immune phenotypes, disease activity, and treatment responsiveness. The *Prevotella* enterotype, in particular, has been associated with favorable responses to MTX (152). These findings emphasize the emerging utility of microbiome-based stratification to tailor RA treatment strategies, mirroring advances seen in other inflammatory diseases such as inflammatory bowel disease (153).

## Conclusions

RA is increasingly recognized as a disease influenced by complex interactions between host immunity and the mucosal microbiome. Accumulating evidence implicates dysbiosis and microbial metabolites in the initiation, progression, and treatment response of RA, particularly through mechanisms such as molecular mimicry, antigen citrullination, immune education, and bacterial translocation. While specific microbial species — *S*. *copri*, *S*. *didolesgii*, *F*. *nucleatum*, *Streptococcus* spp., and others — have been linked to RA pathogenesis, no single microbial trigger has emerged as universally causal. Rather, it is likely that a combination of microbial taxa, mucosal environments, and host genetic susceptibility converge to promote disease. Emerging data on microbial metabolites, including SCFAs, tryptophan catabolites, and bile acids, reveal a dual potential for immunomodulation, offering both insights into pathogenesis and targets for therapy. Additionally, the microbiome may serve as a biomarker to predict disease risk and therapeutic response, particularly to MTX. Despite these advances, critical gaps remain in defining causality, the temporal sequence of microbial shifts relative to autoimmunity, and the efficacy of microbiome-targeted interventions. Future studies will require well-controlled, longitudinal human cohorts, deeper mechanistic understanding, and personalized approaches to harness the microbiome for prevention, early intervention, and individualized treatment in RA.

## Version 1 09/16/2025

Electronic publication
